# Impact of TAVR on coronary artery hemodynamics using clinical measurements and image‐based patient‐specific in silico modeling

**DOI:** 10.1038/s41598-023-31987-w

**Published:** 2023-06-02

**Authors:** Louis Garber, Seyedvahid Khodaei, Nima Maftoon, Zahra Keshavarz-Motamed

**Affiliations:** 1 School of Biomedical Engineering, McMaster University, Hamilton, ON Canada; 2 Department of Mechanical Engineering (Mail to JHE-310), McMaster University, Hamilton, ON L8S 4L7 Canada; 3 Department of Systems Design Engineering, University of Waterloo, Waterloo, ON Canada; 4 Centre for Bioengineering and Biotechnology, University of Waterloo, Waterloo, ON Canada; 5 School of Computational Science and Engineering, McMaster University, Hamilton, ON Canada

**Keywords:** Interventional cardiology, Biomedical engineering

## Abstract

In recent years, transcatheter aortic valve replacement (TAVR) has become the leading method for treating aortic stenosis. While the procedure has improved dramatically in the past decade, there are still uncertainties about the impact of TAVR on coronary blood flow. Recent research has indicated that negative coronary events after TAVR may be partially driven by impaired coronary blood flow dynamics. Furthermore, the current technologies to rapidly obtain non-invasive coronary blood flow data are relatively limited. Herein, we present a lumped parameter computational model to simulate coronary blood flow in the main arteries as well as a series of cardiovascular hemodynamic metrics. The model was designed to only use a few inputs parameters from echocardiography, computed tomography and a sphygmomanometer. The novel computational model was then validated and applied to 19 patients undergoing TAVR to examine the impact of the procedure on coronary blood flow in the left anterior descending (LAD) artery, left circumflex (LCX) artery and right coronary artery (RCA*)* and various global hemodynamics metrics. Based on our findings, the changes in coronary blood flow after TAVR varied and were subject specific (37% had increased flow in all three coronary arteries, 32% had decreased flow in all coronary arteries, and 31% had both increased and decreased flow in different coronary arteries). Additionally, valvular pressure gradient, left ventricle (LV) workload and maximum LV pressure decreased by 61.5%, 4.5% and 13.0% respectively, while mean arterial pressure and cardiac output increased by 6.9% and 9.9% after TAVR. By applying this proof-of-concept computational model, a series of hemodynamic metrics were generated non-invasively which can help to better understand the individual relationships between TAVR and mean and peak coronary flow rates. In the future, tools such as these may play a vital role by providing clinicians with rapid insight into various cardiac and coronary metrics, rendering the planning for TAVR and other cardiovascular procedures more personalized.

## Introduction

Since the first procedure in 2002, transcatheter aortic valve replacement (TAVR) has revolutionized the landscape of interventional cardiology^1^. It has made heart valve replacement accessible to a wider spectrum of patients with aortic stenosis (AS), especially previously inoperable or high-risk populations^1^. Since the initial Food and Drug Administration approval, the number of TAVR surgeries has increased each year and in 2019, TAVR surpassed conventional surgical aortic valve replacement in the United States (72,900 procedures vs. 57,600 respectively)^2^. Similar trends are present globally, with over 450,000 patients in 65 countries undergoing TAVR^3^. However, as is the case with most medical developments, TAVR is associated with some complications and drawbacks. Although the procedure has improved considerably in the past decade, patients still suffer from post-intervention complications such as vascular complications^4^, coronary obstruction^5^, acute coronary syndrome^6^, cerebrovascular events^7^, paravalvular leakage^8^ and others.

Furthermore, a large fraction of patients undergoing TAVR also have comorbid diseases such as coronary artery disease (CAD)^9,10^. Given the high prevalence of concomitant CAD in patients undergoing TAVR (40–70%^11^) and the widespread impact of heart disease and CAD (leading cause of death globally)^12,13^, additional insight into how the procedure would impact coronary blood flow is crucial. Being able to understand, quantify and predict how TAVR would impact coronary blood flow and global hemodynamics on a patient specific basis during the procedure planning may help to prevent adverse coronary related incidences post-TAVR. Acute coronary syndrome for instance, which is caused by a significant reduction in blood flow to the myocardium, has been reported in roughly 5% of patients who underwent TAVR and is associated with a high 30 day morality rate^6^. With rapidly available and quantitative data about coronary hemodynamics, clinicians may be able to better personalize and optimize TAVR planning.

Moreover, as younger and lower risk patients receive TAVR, it is increasingly likely that they will need a follow up valve replacement in their lifetime (valve in valve TAVR for example)^13,14^*.* Recently though, it has become clear that in a substantial number of these cases, invasive coronary catheter access becomes unfeasible due to the leaflet re-location from the first valve implantation^13,14^. Having a tool that could non-invasively simulate coronary blood flow behaviour would allow clinicians to better plan the follow up procedure and screen for possible coronary related complications when invasive access is not possible.

While medical imaging has allowed clinicians to visualize parts of the cardiovascular system, modalities to capture hemodynamics are relatively limited and are usually restricted to larger arteries and ventricles^15^. Furthermore, they are typically limited to imaging velocity instead of blood flow rate and pressure. Angiography (invasive) and CT-angiography (minimally/non-invasive) are the primary imaging methods used to evaluate coronary arteries but are limited to capturing the structure of the vessels^16^. Echocardiography has shown promise in visualizing and quantifying hemodynamics in the coronary arteries but is often limited to just the left main or left anterior descending branch and has seen limited clinical adoption in this domain^17^. Furthermore, it is not possible in all patients and requires extensive technician training to obtain reliable measurements^17^. Recently 4D flow MRI has been applied to capture coronary flow but was limited to only the left main coronary artery and required long scan times^18^. Functional coronary hemodynamic data is predominantly obtained from invasive catheterization to evaluate the severity of CAD and guide coronary interventions, but it is not always collected in the pre/post-TAVR settings^19^.

In the past decade, researchers have paired medical imaging and routine clinical data with the power of computing to generate non-invasive personalized cardiovascular hemodynamics models^20,21^. The marriage of computational science and cardiology has yielded tools capable of simulating possible interventions^22–25^ studying cardiovascular diseases in-silico^26–29^ and generating patient specific metrics^30–33^. While many of these models are aimed at the coronary arteries and compute clinically relevant parameters (such as fractional flow reserve^34,35^), few are designed to simulate or predict the patient-specific impact of TAVR or other non-coronary interventions on coronary hemodynamics. Furthermore, many of these advanced 3D simulation tools require pre-processing and computation time in the order of days for each patient, making the automation and implementation into a clinical workflow challenging^36^. Alternatively, lumped parameter modelling (LPM) offers a simpler, but computationally quicker method to simulate patient specific cardiology models. It relies on using electronic circuits (and the hydraulic-electrical analogy) to simulated waveforms such as blood flow or pressure over time in different regions of the heart^37^. By combining a variety of medical imaging techniques, circuit layouts, element tuning, and optimization techniques, patient-specific waveforms can be obtained^37^. While there exist a series of pure LPMs designed to estimate coronary blood flow^37^, none of them are highly patient specific and utilize multiple clinical modalities to rapidly estimate both cardiac, circulatory and coronary parameters simultaneously. Moreover, none have been directly applied to study patients undergoing TAVR.

In this paper, we developed a novel lumped parameter computational model to simulate blood flow waveforms in the main proximal coronary branches: LAD, LCX and RCA as well as other global cardiovascular hemodynamic parameters. The model was designed to only utilizes limited, non-invasive clinical inputs. The computational model was then applied to 19 patients with AS who underwent TAVR to examine the impact of the procedure on coronary blood flow rate and various cardiovascular metrics. The coronary flow results from the model were compared with those from a patient specific 3D fluid structure interaction (FSI) model (n = 19) along with a model sensitivity analysis.

## Methods

**Figure 1:**
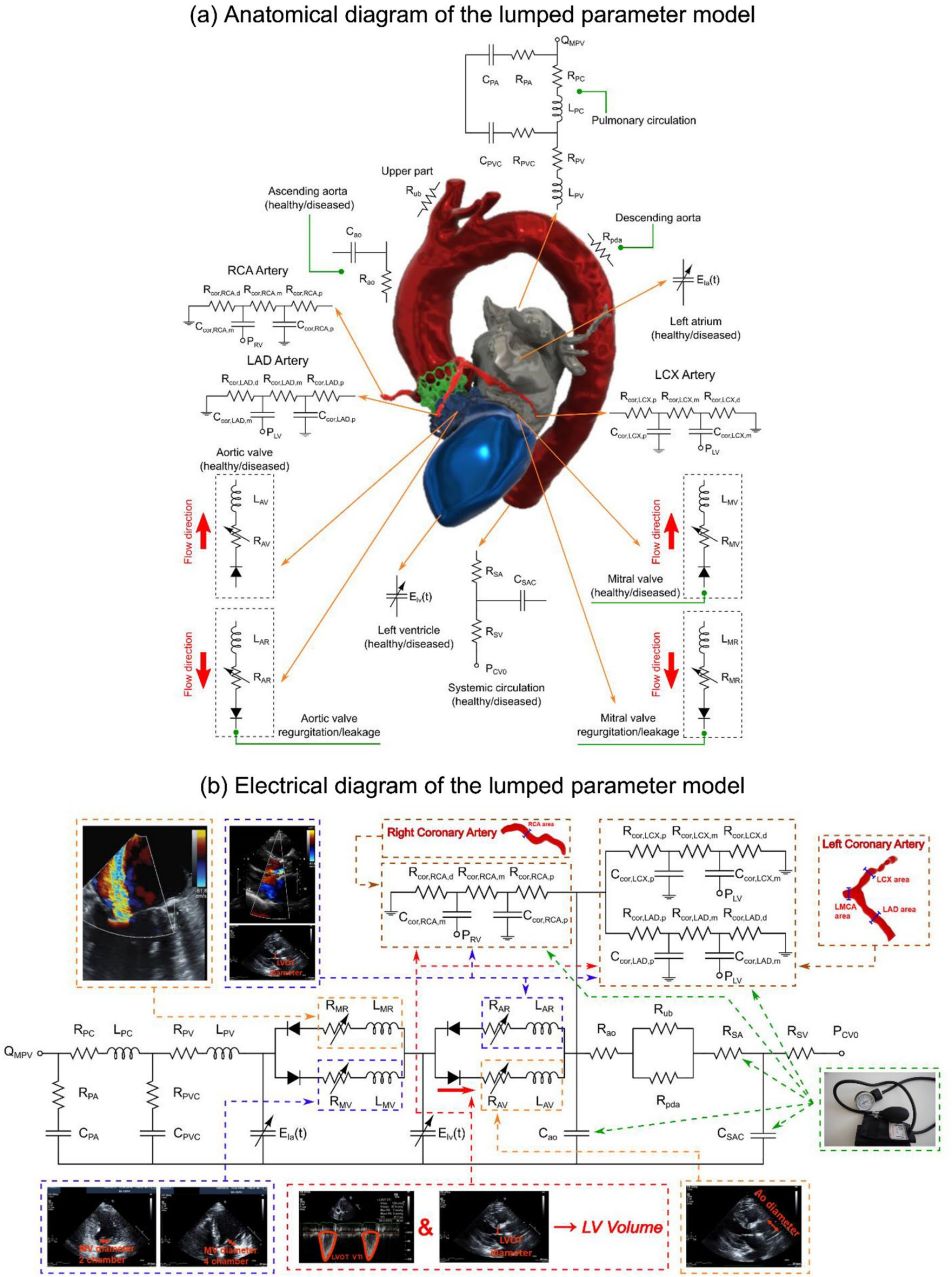
Electrical and anatomical schematic diagrams of the LPM. (**a**) Anatomical illustration showing the different circuit meshes and their relationship to the cardiovascular system; (**b**) Electrical diagram with data inputs. The model includes the following sub-models: LAD, LCX and RCA, left ventricle, aortic valve, left atrium, mitral valve, aortic valve regurgitation, mitral valve regurgitation, systemic circulation, pulmonary circulation. Abbreviations in the schematic are the same as in Table 1.

A novel, proof-of-concept patient-specific, image-based LPM was developed, validated, and tested in this study (Fig. 1, Schematic Diagram; Table 1). The model was aimed at: (1) quantifying metrics of circulatory function (global hemodynamics); (2) quantifying metrics of cardiac function (global hemodynamics); (3) providing non-invasive insight into coronary blood flow patterns in the pre-intervention and in the post-intervention states (local hemodynamics). The computational model used Doppler echocardiography (DE), computed tomography (CT) and sphygmomanometer data to generate patient-specific cardiovascular models. The developed computational model was tested on a retrospective dataset of 19 patients who underwent transcatheter aortic valve replacement (TAVR). The aim was to quantify the impact of the procedure on circulatory, cardiac and coronary artery blood flow metrics without the use of invasive catheters.

**Table 1:** Parameter summary for patient specific LPM.

Parameter description	Abbreviation	Value
Valve parameters		
Effective orifice area	EOA	Measured using DE
Energy loss coefficient	E_L_CO	$\frac{\left(EAO\right)A}{A-EOA}$; A measured using DE
Mitral valve inertance	M_MV_	Constant value: 0.53 g/cm^2,38^
Systemic circulation parameters		
Aortic resistance	R_ao_	Constant value: 0.05 mmHg s/mL^41^
Aortic compliance	C_ao_	Initial value: 0.5 mL/mmHg Optimized based on branchial pressure
Systemic vein resistance	R_SV_	Constant value: 0.05 mmHg s/mL^41^
Systemic arteries and veins compliance	C_SAC_	Initial value: 2 mL/mmHg Optimized based on branchial pressure
Systemic arteries resistance	R_SA_	Initial value: 0.8 mmHg s/mL Optimized based on branchial pressure
Upper body resistance	R_ub_	Adjusted to direct 15% of total flow rate in healthy cases^53^
Proximal descending aorta resistance	R_pda_	Constant value: 0.05 mmHg s/mL^41^
Elastance function parameters		
Maximum elastance	E_max_	2.1 (LV)|0.17 (LA)^39,40^
Minimum elastance	E_min_	0.06 (LV and LA)^39,40^
Elastance ascending gradient	m_1_	1.32 (LV and LA)^39,40^
Elastance descending gradient	m_2_	27.4 (LV)|13.1 (LA)^39,40^
Elastance ascending time translation	$\tau$_1_	0.269 T (LV)|0.110 T (LA)^39,40^
Elastance descending time translation	$\tau$_2_	0.452 T (LV)|0.18 T (LA)^39,40^
Coronary parameters		
Proximal coronary resistance	R_cor,p_	Adjusted based on CO, MAP, and coronary branch cross sectional area
Medial coronary resistance	R_cor,m_	Adjusted based on CO, MAP, and coronary branch cross sectional area
Distal coronary resistance	R_cor,d_	Adjusted based on CO, MAP, and coronary branch cross sectional area
Proximal coronary compliance	C_cor,p_	Adjusted based on total coronary compliance and branch cross sectional area
Medial coronary compliance	C_cor,m_	Adjusted based on total coronary compliance and branch cross sectional area
Pulmonary circulation parameters		
Pulmonary vein inertance	L_PV_	Constant value: 0.0005 mmHg s^2^/mL^38^
Pulmonary vein resistance	R_PV_	Constant value: 0.002 mmHg s/mL^38^
Pulmonary vein and capillary resistance	R_PVC_	Constant value: 0.001 mmHg s/mL^38^
Pulmonary vein and capillary compliance	C_PVC_	Constant value: 40 mL/mmHg^38^
Pulmonary capillary inertance	L_PC_	Constant value: 0.0003 mmHg s^2^/mL^38^
Pulmonary capillary resistance	R_PC_	Constant value: 0.21 mmHg s/mL^38^
Pulmonary arterial resistance	R_PA_	Constant value: 0.01 mmHg s/mL^38^
Pulmonary arterial compliance	C_PA_	Constant value: 4 mL/mmHg^38^
Mean flow rate of pulmonary valve	Q_MPV_	Optimized flow parameter s.t the model could reproduce the Forward LVOT-SV seen in DE
Input and output conditions		
Forward left ventricular outflow tract stroke volume	Forward LVOT-SV	Measured using DE
Central venous pressure	P_CV0_	Constant value: 4 mmHg^41^
Additional parameters		
Heart rate	HR	Measured using DE
Duration of cardiac cycle	T	Measured using DE
Density of blood	$\rho$	Constant value: 1050 kg/m^3,41^
Systolic end ejection time	T_EJ_	Measured using DE
End diastolic volume	EDV	Measured using DE
End systolic volume	ESV	Measured using DE

Our lab previously developed a non-invasive diagnostic computational-mechanics framework for complex valvular, vascular and ventricular disease (called C3V-LPM for simplicity)^41^. The method was described in detail elsewhere^41^. In this study, we further developed C3V-LPM to enable the quantification of local and global hemodynamics in patients with mixed and complex valvular, vascular, mini-vascular and ventricular diseases (known as C3VM-LPM) (Fig. 1, Table 1). The developed computational model uses limited input parameters that can all be reliably measured non-invasively using DE, CT and a sphygmomanometer. Currently, none of the above metrics (global and local hemodynamics) can be obtained noninvasively in patients and when invasive procedures are performed, the gathered metrics cannot be by any means as complete as the results that C3VM-LPM provides. The previously created model, C3V-LPM, was validated against clinical catheterization data in forty-nine AS patients with a substantial inter- and intra-patient variability with a wide range of disease^41^. In addition, some of the sub-models of the patient-specific LPM algorithm have been used and validated previously^30,31,42–52^, with validation against in vivo cardiac catheterization^53,54^ in patients with vascular diseases, in vivo MRI data^55^ in patients with AS, and in vivo MRI data^56–58^ in patients with coarctation and mixed valvular diseases.

The major development with the new C3VM-LPM is the additional capability to non-invasively capture and quantify patient-specific hemodynamics in the following left and right coronary artery branches: (1) LAD, (2) LCX, (3) RCA. The following sections outline the different compartments and tuning approaches developed for this patient specific model (see Fig. 1 for the complete electrical representation).

### Study population and data acquisition

19 patients who underwent TAVR in 2020 at St. Joseph's Healthcare and Hamilton Health Science (Hamilton, Canada) were considered in this study. The study protocols were reviewed and approved by the Hamilton Integrated Research Ethics Board (HiREB) for Hamilton Health Science and St. Joseph’s Healthcare. Informed consents were obtained from all human participants. All methods and measurements were performed in accordance with all relevant guidelines and regulations including guidelines from the American College of Cardiology and American Heart Association. Data was collected at 2 time points: pre-procedure and post-procedure. Table 2 outlines the demographic and procedural data of the patients. All data and results are expressed as mean ± standard deviations (SD).

**Table 2:** Baseline and post-TAVR patient characteristics.

	Pre-TAVR (n = 19, mean ± SD)	90-day post-TAVR (n = 19, mean ± SD)
Patient characteristics		
Age (year)	77.8 ± 6.0	N/A
Female subjects	10 (53%)	N/A
Mean weight (kg)	85.2 ± 33.3	N/A
Mean height (cm)	168.5 ± 9.6	N/A
Body mass index (kg/m^2^)	2.0 ± 0.4	N/A
NYHA—Class I	0	11 (58%)
NYHA—Class II	10 (53%)	3 (16%)
NYHA—Class III	9 (47%)	5 (26%)
NYHA—Class IV	0	0
Arterial characteristics		
Brachial systolic BP (mmHg)	133.0 ± 18.9	142.0 ± 22.3
Brachial diastolic BP (mmHg)	70.5 ± 9.2	72.0 ± 15.4
Hypertension	15 (79%)	N/A
Coronary artery disease	5 (26%)	N/A
Echocardiography findings		
Heart rate (bpm)	71 ± 14	73 ± 13
Ejection fraction (%)	59.9 ± 8.4	62.3 ± 7.0
Stenotic aortic valve EOA (cm^2^)	0.84 ± 0.19	N/A
Stenotic aortic valve type	Tricuspid: 11 (58%)	N/A
	Bicuspid: 5 (26%)	
	Unknown: 3 (16%)	
Max aortic valve flow velocity (m/s)	4.45 ± 0.56	2.75 ± 0.65
Mean aortic valve pressure gradient (mmHg)	47.2 ± 13.1	18.2 ± 8.3
Paravalvular leakage	N/A	Trace: 0
		Mild: 1
		Moderate-to-severe: 1
		Severe: 0

### Coronary arteries

Each of coronary branches is modeled using a circuit comprised of 3 resistors ($${\mathrm{R}}_{\mathrm{cor},\mathrm{p}},{\mathrm{R}}_{\mathrm{cor},\mathrm{ m}},{\mathrm{R}}_{\mathrm{cor},\mathrm{d}})$$, 2 capacitors ($${\mathrm{C}}_{\mathrm{cor},\mathrm{p}}, {\mathrm{C}}_{\mathrm{cor},\mathrm{ m}})$$ and an embedded pressure (voltage) source ($${P}_{im})$$. This circuit representation was initially proposed by Mantero et al.^59^ and further advanced and popularized by Kim et al.^60^. It has been used in numerous LPMs^34,61–64^ and has been shown to capture the bi-phasic nature of coronary flow, in which peak blood flow occurs during the diastole phase rather than during systole^59,60^. While inductors are including in the ventricle and valvular portion of the model, they were not included in the coronary branches since the inertial phenomena is not significant in the coronary arteries^59^. The following ODEs are obtained from the circuit layout to model each of the coronary branches^63^:

1 $${\mathrm{q}}_{\mathrm{in}}= \frac{{\mathrm{P}}_{\mathrm{in}}- {\mathrm{P}}_{\mathrm{p}}}{{\mathrm{R}}_{\mathrm{cor},\mathrm{p}}}$$

2 $${\mathrm{q}}_{\mathrm{in}}={\mathrm{C}}_{\mathrm{cor},\mathrm{p}}\frac{{\mathrm{dP}}_{\mathrm{p}}}{\mathrm{dt}}+{\mathrm{q}}_{\mathrm{m}}$$

3 $${\mathrm{P}}_{\mathrm{p}}={\mathrm{q}}_{\mathrm{m}}{\mathrm{R}}_{\mathrm{cor},\mathrm{m}}+{\mathrm{P}}_{\mathrm{m}}$$

4 $${\mathrm{q}}_{\mathrm{m}}={\mathrm{q}}_{\mathrm{out}}+{\mathrm{C}}_{\mathrm{cor},\mathrm{m}}\frac{{\mathrm{dP}}_{\mathrm{im}}}{\mathrm{dt}}$$

5 $${\mathrm{P}}_{\mathrm{m}}={\mathrm{q}}_{\mathrm{out}}{\mathrm{R}}_{\mathrm{cor},\mathrm{d}}+{\mathrm{P}}_{\mathrm{out}}$$

where ${\mathrm{q}}_{\mathrm{in}}$, ${\mathrm{P}}_{\mathrm{in}}, {\mathrm{q}}_{\mathrm{out}}$ and ${\mathrm{P}}_{\mathrm{out}}$ are the blood flow and pressure into and out of the coronary branch. ${\mathrm{R}}_{\mathrm{cor},\mathrm{p}},{\mathrm{R}}_{\mathrm{cor},\mathrm{m}},{\mathrm{R}}_{\mathrm{cor},\mathrm{d}}$ are the proximal, medial, and distal resistors while ${\mathrm{C}}_{\mathrm{cor},\mathrm{p}}, {\mathrm{C}}_{\mathrm{cor},\mathrm{m}}$ are the proximal and medial capacitors. ${\mathrm{P}}_{\mathrm{p}}$, ${\mathrm{P}}_{\mathrm{m}}$ and ${\mathrm{P}}_{\mathrm{im}}$ are the proximal, medial and intramyocardial pressures.

${\mathrm{P}}_{\mathrm{im}}$ is set to be either the left ventricle (LV) or right ventricle (RV) pressure, depending on the coronary artery that it is coupled to. In this study, we used the LV pressure for the left branches (LAD and LCX) and 0.5P_LV_^32^ to create the RV pressure for the right branch (RCA).

#### Determining arterial resistance and compliance in coronaries

##### Total coronary resistance

The mean flow rate to the coronary arteries was assumed to be 4.0% of the cardiac output (CO)^60^. The total coronary resistance was then estimated based on a relationship between pressure and flow^34^:

6 $${R}_{cor,total}=\frac{MAP}{{Q}_{cor,total}}=\frac{MAP}{\left(0.04\right)*CO}$$

where ${R}_{cor,total}$ is the total coronary resistance and mean arterial pressure (MAP) is calculated based on systolic blood pressure (SBP), diastolic blood pressure (DBP) and heart rate (HR)^65^:

7 $$MAP=DBP+[\frac{1}{3}+\left(HR*0.0012\right)](SBP-DBP)$$

##### Coronary vessel resistance and compliance

The total coronary resistance was divided between each of the branches based on a variation of Murray’s law^66^, which relates resistance to vessel diameter:

8 $${R}_{cor,j}= \frac{\sum_{i=1}^{n}{\sqrt{{A}_{i}}}^{2.6}}{{\sqrt{{A}_{j}}}^{2.6}} {R}_{cor,total } \quad where\, j=\{LAD,\,LCX\, or\, RCA\}$$

where ${R}_{cor,j}$ is the total coronary resistance in the desired branch and ${A}_{i}$ is the cross sectional area of each of the coronary vessels^60^. Further division of the total vessel resistance into the 3 resistive elements in the circuit was based on the work of Sankaran et al.^67^:

9 $${R}_{cor,j,p }= {(0.32)R}_{cor,j } \quad {R}_{cor,j,m }= {(0.52)R}_{cor,j} \quad {R}_{cor,j,d }= (0.16){R}_{cor,j}$$

where ${R}_{cor,j,p }{, R}_{cor,j,m }{, R}_{cor,j,d}$ are the proximal, medial, and distal resistors.

To account for the cases with coronary vessel stenoses or vessels with considerable reductions in diameters, the following approach was used^68^:

10 $$\alpha =\frac{{A}_{sten}}{{A}_{0}}$$

11 $${R}_{cor, red, j}={R}_{cor,j}{(\alpha }^{-2})$$

where $${A}_{sten}$$ represents the cross-sectional area of the stenosis/diameter reduction and $${A}_{0}$$ represents the normal cross-sectional area (non-stenotic area). The original resistance for the vessel ($${R}_{cor, j}$$), assuming no stenosis, is then multiplied with an area reduction factor ($$\alpha$$) to yield the new branch resistance ($${R}_{cor, red, j}),$$ which can then be further divided into the sub resistors.

The left coronary compliance was computed by dividing up the total left coronary compliance based on vessel diameter:

12 $${C}_{cor,j}= \frac{{A}_{j}}{\sum_{i=1}^{n}{A}_{i}} {C}_{cor,total }^{L}$$

where ${C}_{cor,j}$ is the left coronary vessel compliance, ${C}_{cor,total }^{L}$ is the total left coronary compliance and ${A}_{i}$ is the cross sectional area of each of the left coronary branches^60^. A manual tuning process was utilized to determine total left coronary compliance value that lead to physiological coronary flow waveforms^69–71^.

The compliances were then divided across the 2 capacitors based on the following relationship, developed by Sankaran et al.^67^:

13 $${C}_{cor,j,p}=(0.11){C}_{cor,j} \quad {C}_{cor,j,m}=(0.89){C}_{cor,j}$$

where ${C}_{cor,j,p}$ and ${C}_{cor,j,m}$ are the proximal and medial capacitors. The same process was applied for the right coronary vessels.

#### Input parameters and geometry reconstruction

The C3VM-LPM used the following patient specific measurements as inputs: forward left ventricle outflow tract stroke volume (Forward LVOT-SV), cardiac cycle time (T), ejection time (T_EJ_), effective orifice area of the aortic valve ($EO{A}_{AV}$), effective orifice area of the mitral valve ($EO{A}_{MV}$), area of left ventricle outflow tract (${A}_{LVOT}$), aortic regurgitant effective orifice area ($EO{A}_{AR}$), mitral regurgitant effective orifice area ($EO{A}_{MR}$) and paravalvular leakage volume (${V}_{leak}$) measured by DE. Branchial systolic and diastolic blood pressure were measured by a sphygmomanometer.

ITK-SNAP (version 3.8.0-BETA)^72^ and the collected CT data were used to re-construct the 3D geometries of the main coronary arteries (left main coronary artery (LMCA), proximal LAD, LCX and RCA) in both the pre-TAVR and post-TAVR cases. Figure 1b outlines how the inputs parameters are related to the lumped parameter sub-models.

#### Computational algorithm

The ordinary differential equations which govern the LPM circuit were formulated and solved in Matlab Simscape (MathWorks Inc, Natick USA). Addition functions were written in Matlab and Simulink to enhance the Simscape code. The Matlab Optimization Toolbox and Simulink Design Optimization Toolbox were also used to implement part of the parameter tuning algorithms based on in-house code. The trapezoid rule variable step solver (ode23t) was used with an initial step time of 0.1 ms. The initial voltages and currents of the capacitors and inductors in the circuit were set to zero and the convergence residual criterion was set to 10^–6^. On average, the model had a computation time in the order of 10–15 s (on a workstation with the configurations of Intel Core*™* i7-10700 CPU @2.90 GHz and 64 GB Ram). Table 3 outlines all the model parameters and their values or formulas.

**Table 3:** Maximum relative error (%) in the computed mean coronary branch flow rates from the sensitivity analysis in response to independent variation in model parameters and inputs.

Description	Parameter	Range	Max relative error – LAD (%)	Max relative error – LCX (%)	Max relative error – RCA (%)	Mean across LAD/LCX/RCA (%)
Mean arterial pressure	MAP	± 20%	28.4	27.9	25.7	27.4
Cardiac output	CO	± 20%	22.9	22.4	20.8	22.0
LAD area	A_LAD_	± 20%	16.0	13.5	13.0	14.2
LCX area	A_LCX_	± 20%	7.3	21.1	9.3	12.6
RCA area	A_RCA_	± 20%	7.1	7.2	20.2	11.5
Total left coronary compliance	C^L^_cor,total_	± 20%	5.2	5.0	–	5.1
Total right coronary compliance	C^R^_cor,total_	± 20%	–	–	0.7	0.7

## Results

### Model verification

In many cases, during the pre-TAVR workup, invasive flow and pressure data in the coronary arteries are not collected and angiography images are often used to decide if the coronary arteries should be re-vascularized before, during or after TAVR^19^. Since this invasive coronary data in the pre- and post-TAVR cases is limited and not routinely collected, we used our patient-specific 3D FSI model results to validate our newly developed LPM. While this approach is not true gold-standard validation, using a complex FSI model offers a strong proof-of-concept verification method to examine the performance of the LPM. This full 3D modelling technique was applied to the 19 patients and the mean and peak flows for the LAD, LCX and RCA were computed.

The 3D FSI model used individual CT images to reconstruct the geometry of the coronary arteries, proximal ascending aorta, and aortic valve leaflets. Our lab previously developed a non-invasive diagnostic computational-mechanics framework for complex valvular, vascular and ventricular disease (called C3V-LPM for simplicity)^41^. The method was described in detail elsewhere^41^. In this study, we further developed C3V-LPM to enable the quantification of local and global hemodynamics in patients with mixed and complex valvular, vascular, mini-vascular and ventricular diseases (known as C3VM-LPM) (Fig. 1). Boundary conditions were obtained from C3VM-LPM (Fig. 1) to provide the ascending aorta and left ventricle pressure waveforms. The FSI interface wall was defined as the interface between the coronary arteries walls and the tissue as the solid domain (please see the supplementary Fig. S1). The 3D coronary arteries flow was simulated using FSI method^73–75^ using finite volume method—the details of FSI algorithm can be found elsewhere^22,23,76^. Due to the complexity of heart valve motions during full cardiac cycle, the FSI model simulated blood flow in the structure during the diastole phase (main filling phase for coronaries) assuming rigidly closed aortic valve. As the majority of coronary blood flow occurs in diastole (due to the impact of extravascular ventricle compression in systole^77^), this allows for a relatively complete validation of the total blood flow during the cardiac cycle. The patient-specific lumped parameter model (C3V-LPM) and 3D FSI modelling was previously validated against in-vivo Doppler echocardiography data as explained in Khodaei et al.^22,23^ and Keshavarz-Motamed et al.^30^.

**Figure 2:**
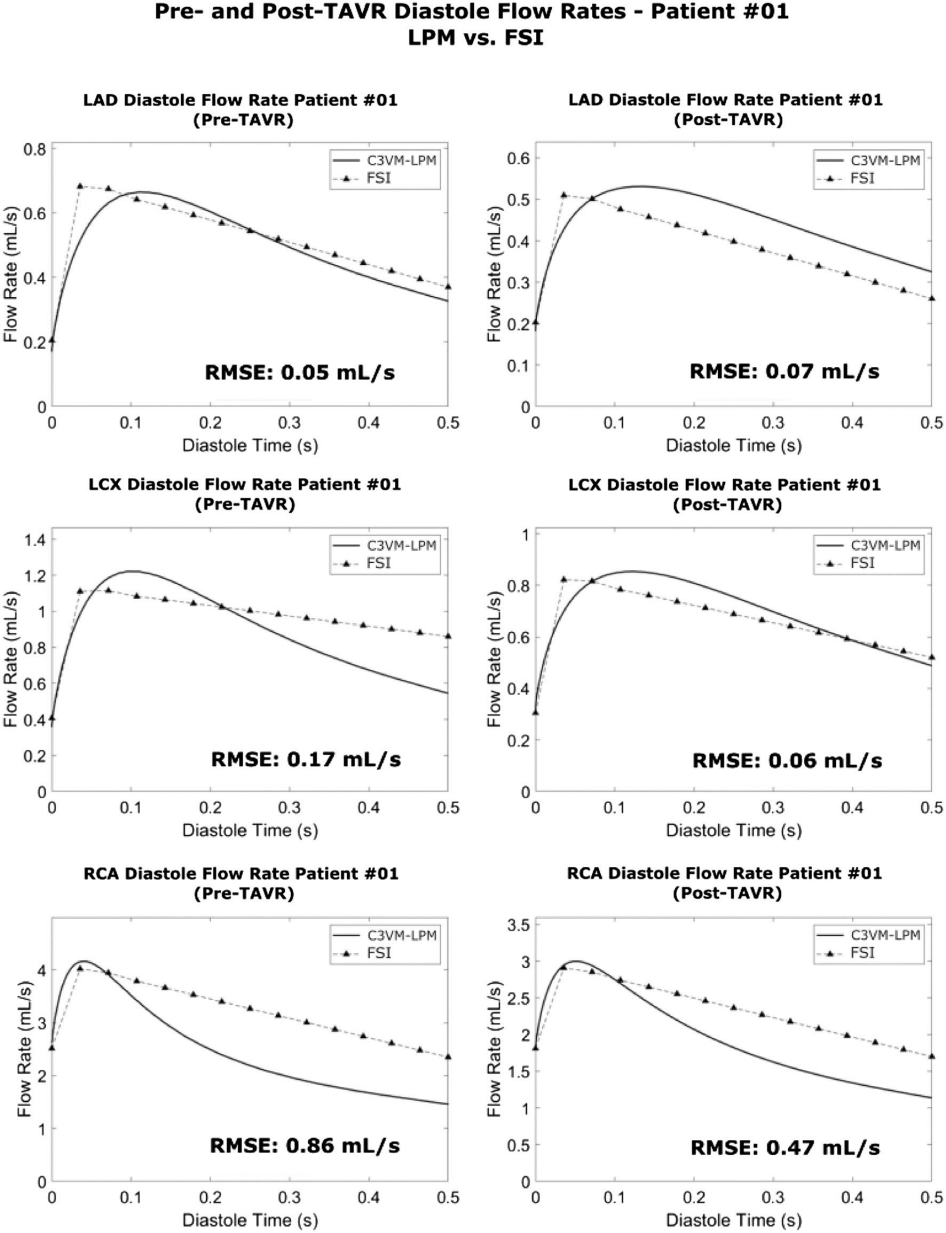
Coronary blood flow waveform validation—Patient #01. The pre- and post-TAVR diastole blood flow waveforms in all 3 branches (LAD, LCX and RCA) from the LPM and the 3D FSI model for patient #01. The time has been normalized to 0.5 s. RMSE—root mean squared error between the waveforms.

**Figure 3:**
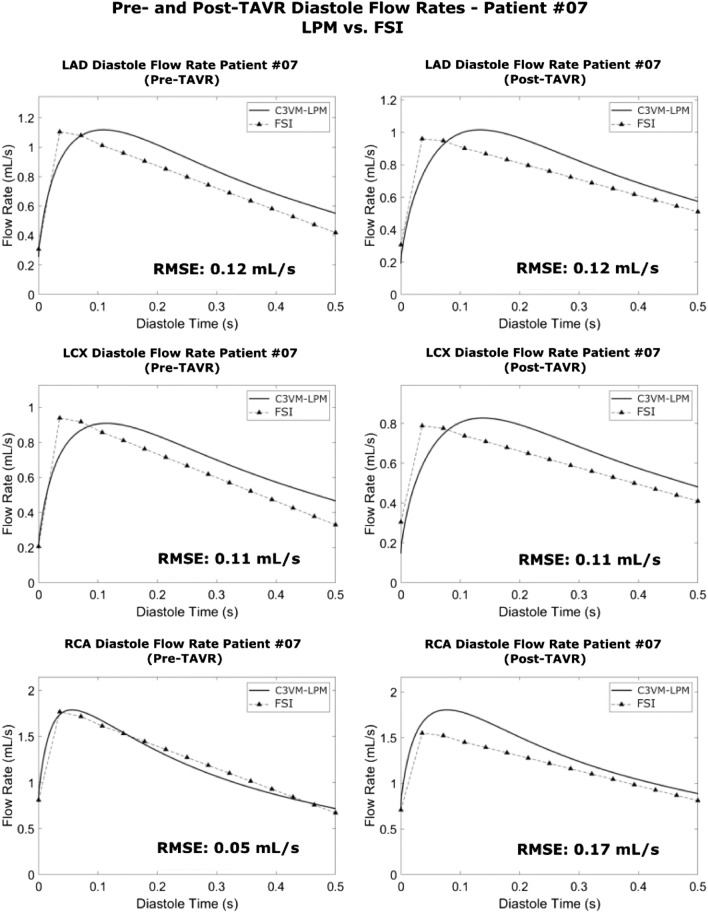
Coronary blood flow waveform validation—Patient #07. The pre- and post-TAVR diastole blood flow waveforms in all 3 branches (LAD, LCX and RCA) from the LPM and the 3D FSI model for patient #07 The time has been normalized to 0.5 s. RMSE—root mean squared error between the waveforms.

Figures 2 and 3 outline the blood flow waveforms in the pre- and post-TAVR settings for all 3 coronary branches for two samples patients according to the LPM developed in this paper (C3VM-LPM) along with the 3D FSI model results. Overall, there is a strong agreement in the waveforms between the modelled coronary blood flow rates from the CV3M-LPM (lumped) and the FSI (3D) model. Table 4 outlines the average mean and peak flow rate error between the two models in pre- and post-TAVR (n = 19).

**Table 4:** Mean and peak blood flow rate error % (± std) between the LPM and the FSI models in the three main coronary artery branches (n = 19).

	Mean flow rate error			Peak flow rate error		
	LAD	LCX	RCA	LAD	LCX	RCA
Pre-TAVR	13.2 ± 17%	11.7 ± 11%	16.1 ± 29%	15.3 ± 14%	18.1 ± 18%	22.7 ± 28%
Post-TAVR	17.3 ± 17%	11.0 ± 15%	13.3 ± 10%	15.9 ± 19%	15.2 ± 24%	19.9 ± 18%

To better understand how the C3VM-LPM responded to possible independent variations in parameters and inputs, a sensitivity analysis was conducted. The focus of this analysis was on the coronary branches as previous parameter analyses have been conducted on the values in the cardiac and circulatory regions; see^42,43^ and^46^ for more details. Table 3 outlines the parameters that control the mean flow rate and shape of the coronary flow curves in the model (see Eqs. 6–12). Each parameter was independently varied by ± 20% and the maximum relative error percentage in the computed mean flow rate for the LAD, LCX and RCA was tabulated (Table 3). Following the approach of Tran et al.^78^, the heart rate was assumed to a deterministic parameter.

Table 3 outlines the results from the coronary branch sensitivity analysis. The mean coronary flow rates estimated from the model are relatively sensitive to changes in MAP (27.4% max relative error averaged across all 3 branches), CO (22.0%) and branch cross sectional area (LAD—14.2%, LCX—12.6% and RCA—11.5%). Conversely, the mean coronary flow rate is not significantly impacted by changes in the left and right coronary compliance (5.1% and 0.7% respectively). Vessel compliance tends to impact the shape the waveform rather than the mean flow rate directly^78^. When the left and right coronary compliances were varied by ± 20%, the max relative error in the peak flow rates were only 7.1% and 7.4% respectively.

In addition to above mentioned analysis, we performed another sensitivity analysis for coronary diameter to determine the possible impact of segmentation error on the predicted coronary blood flow from the LPM model. In the analysis, a post-TAVR subject without CAD was selected (Patient #12) to emphasize the impact of segmentation error. The diameter of the LCX branch (originally measured at 1.5 mm) was varied by ± 0.5 mm and the impact on the predicted coronary flow rate in all the 3 coronary branches was tabulated (Table S1). In the case of the mean LCX blood flow, there is a proportional response between diameter and flow while there is an inversely proportional response in the other two branches. Since the blood flow to coronary arteries is relatively constant (~ 4% of the cardiac output), if the diameter of one coronary vessel is increased, flow in this branch will also increase, leading to decreased blood flow in the other branches.

### Cardiac and circulatory function and hemodynamics (global hemodynamics)

**Figure 4:**
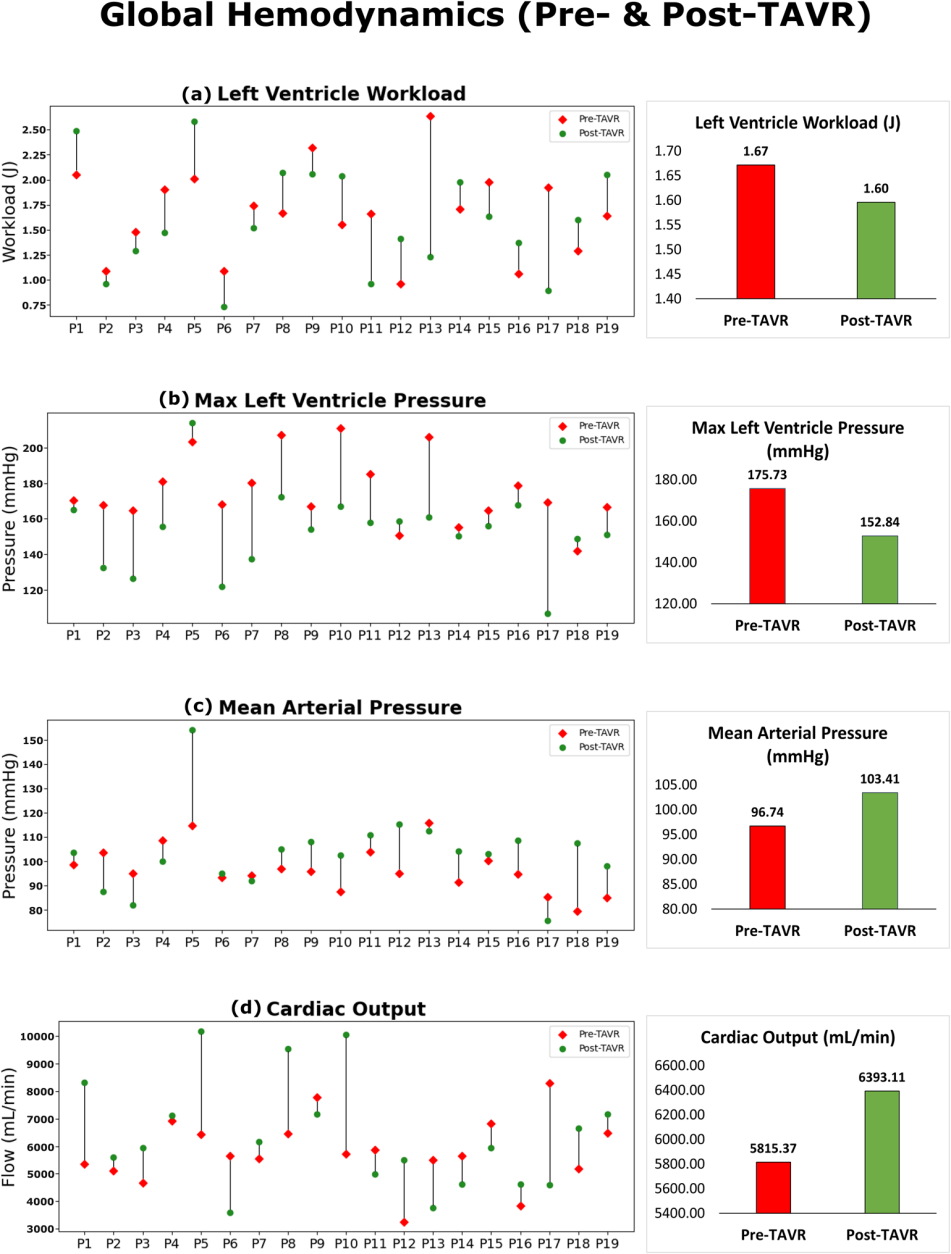
Global hemodynamic metrics pre- and post-TAVR. The changes in individual and mean global hemodynamic metrics from pre-TAVR to post-TAVR (n = 19) for (**a**) left ventricle workload (J); (**b**) max left ventricle pressure (mmHg); (**c**) mean arterial pressure (mmHg); (**d**) cardiac output (mL/min).

Using the lumped model, pre- and post-TAVR cardiac and ventricular indices were calculated for the patients (Fig. 4). All the patients who underwent the procedure had aortic stenosis and the severity was assessed by senior cardiologists based on aortic valve flow dynamics according to the European Association of Cardiovascular Imaging and American Society of Echocardiography guidelines^79^.

The reduction in valve area caused by aortic stenosis led to the formation of high velocity jets driven by the pressure gradient across the valve. In all but 1 patient, valve pressure gradient decreased (61.5% on average) after TAVR. While the valvular pressure reduced for almost all patients, this reduction is not always associated with improved global hemodynamics and better prognosis^30^.

LV workload is a measure of the required work by the left ventricle to eject blood and overcome the opposing cardiovascular systemic load^22,55^. The workload was computed through the integral of the left ventricle pressure–volume loop generated by the lumped model. On average, the workload decreased by 4.5% after TAVR (but increased in 9 of the 19 subjects). Similarly, the presence of aortic stenosis pre-TAVR led to elevated LV pressure and impaired LV function for the patients. By surgically implanting the valve, TAVR led to the reduction in LV pressure for 16 of the 19 patients and decreased the pressure by 13.0% on average.

SBP increased in 13 patients (7.3% average increase across all patients) and DBP increased in 13 patients (5.5% average increase). Mean atrial pressure, which represents a weighted average between SBP and DBP, increased by 6.9% on average after TAVR, while increasing for 13 of the patients. Sustained increase in blood pressure after TAVR is often associated with better prognosis while decreased BP may be linked to less favourable prognoses^80,81^. Similarly, cardiac output increased by 9.9% on average (increased in 12 patients) and a 1.9% increase in resting heart rate was observed (increase in 12 patients—not shown in Fig. 4).

### Coronary blood flow dynamics (local hemodynamics)

Coronary blood flow is crucial for delivering oxygen to the myocardium and is heavily governed by numerous physiological factors including cardiac output, heart rate, ventricular pressure, coronary perfusion pressure, vessel diameter, aortic valve area, disease status (such as AS or CAD) as well as other biological regulation factors^82,83^.

**Figure 5:**
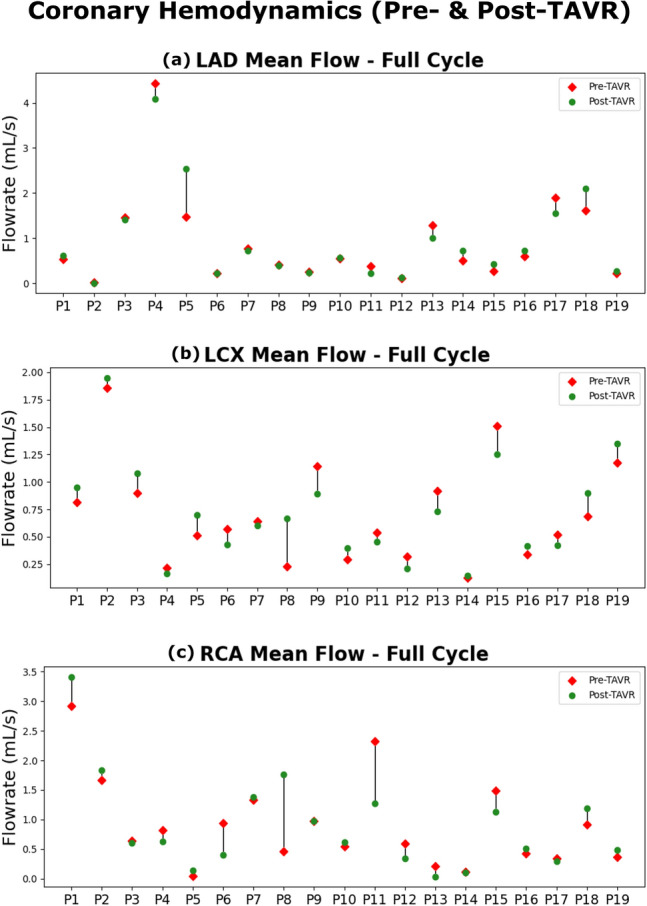
Coronary blood flow rate pre- and post-TAVR. The changes in individual coronary blood flow rate (mL/s) from pre-TAVR to post-TAVR (n = 19) (**a**) LAD; (**b**) LCX; (**c**) RCA.

As there was patient-specific variation in many of these parameters (Fig. 4), there was also large individual variations in the impact of TAVR on coronary artery blood flow across the 3 branches (Fig. 5). Of the 19 patients, 7 had increases in coronary blood flow in all branches, 6 patients had decreases in all branches, while the remainder had increases and decreases in difference branches. Across all the patients, mean flow increased by 2.8% on average post-TAVR (5.4%, − 3.0% and − 0.1% for the LAD, LCX and RCA branches, respectively; N = 19). When broken down into the cardiac phases, the coronary blood flow increased by 17.5% post-TAVR during systole (15.7%, 22.6% and 14.3% for the LAD, LCX and RCA branches, respectively; N = 19) while decreasing by 7.1% during diastole (− 1.2%, − 7.6% and − 12.4% for LAD, LCX and RCA branches; N = 19).

Similar trends were observed for the peak coronary flow rate. After TAVR, there was an 8.4% increase during systole (3.8%, 6.8% and 14.5% LAD, LCX and RCA branches; N = 19) but a 11.1% decrease during diastole (-5.1%, -11.8% and -16.3% LAD, LCX and RCA branches; N = 19). Overall, we observed that the mean coronary blood flow rate was predominantly impacted during systole, whereas the peak coronary blood flow rate was primarily influenced during diastole.

#### Patient specific coronary hemodynamics

Coronary artery blood flow dynamics are impacted by various physiological factors and diseases^82^. For a computational tool to appropriately predict these waveforms, it must be able to capture the patient specific impacts between the various cardiac and circulatory interactions. Since the C3VM-LPM is designed to include not only the coronary arteries but also the pulmonary circulation, left atrium, mitral valve, aortic valve, ascending aorta and systemics circulation, it can simulate a portion of the cardiovascular system. Furthermore, it can provide a window to examine various aspects of the system in both the pre- and post-intervention setting for individual patients.

**Figure 6:**
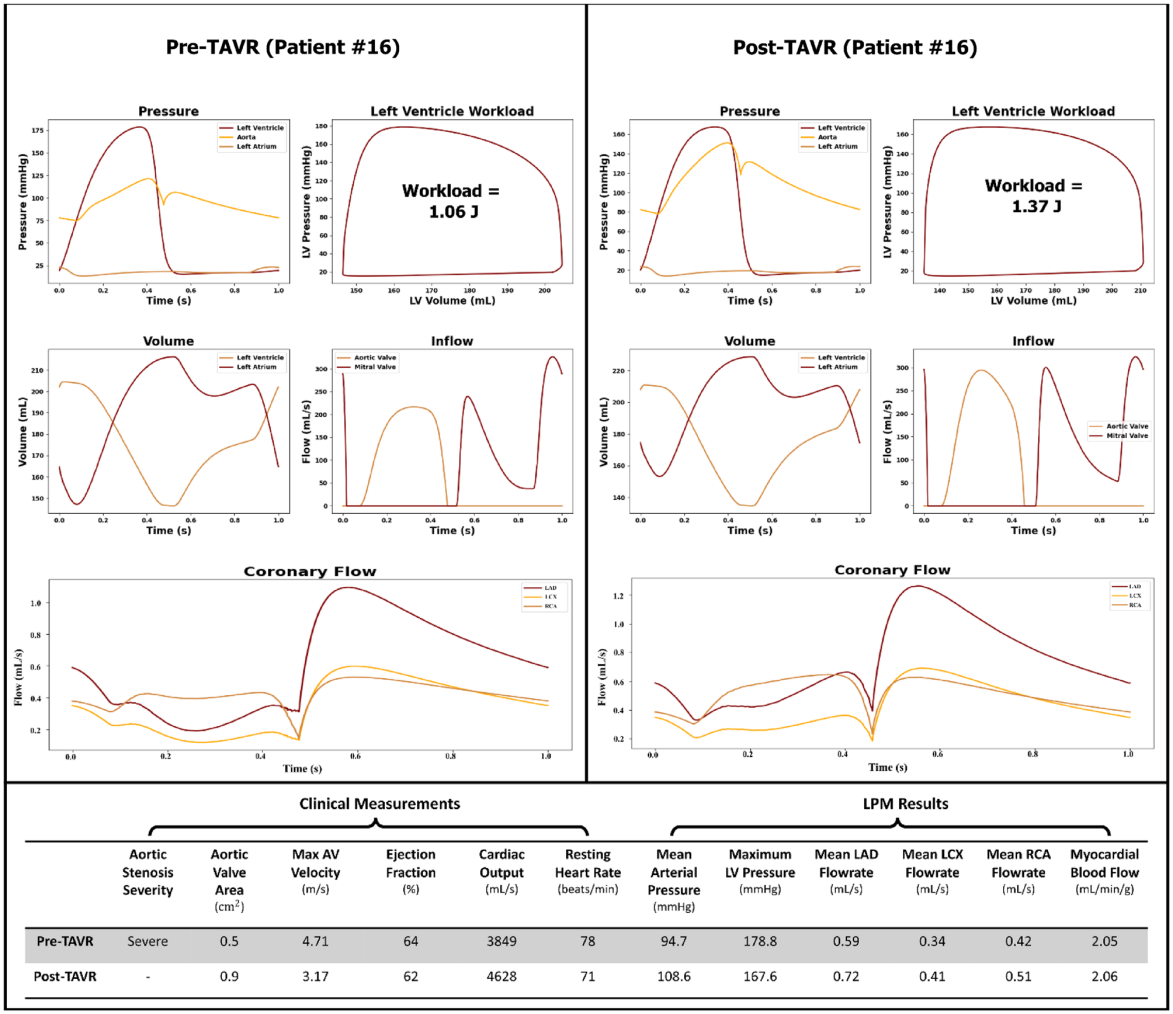
Predicted cardiac and coronary hemodynamics (Patient #18). The plots on the left and right illustrate the pre-TAVR and post-TAVR data respectively.

**Figure 7:**
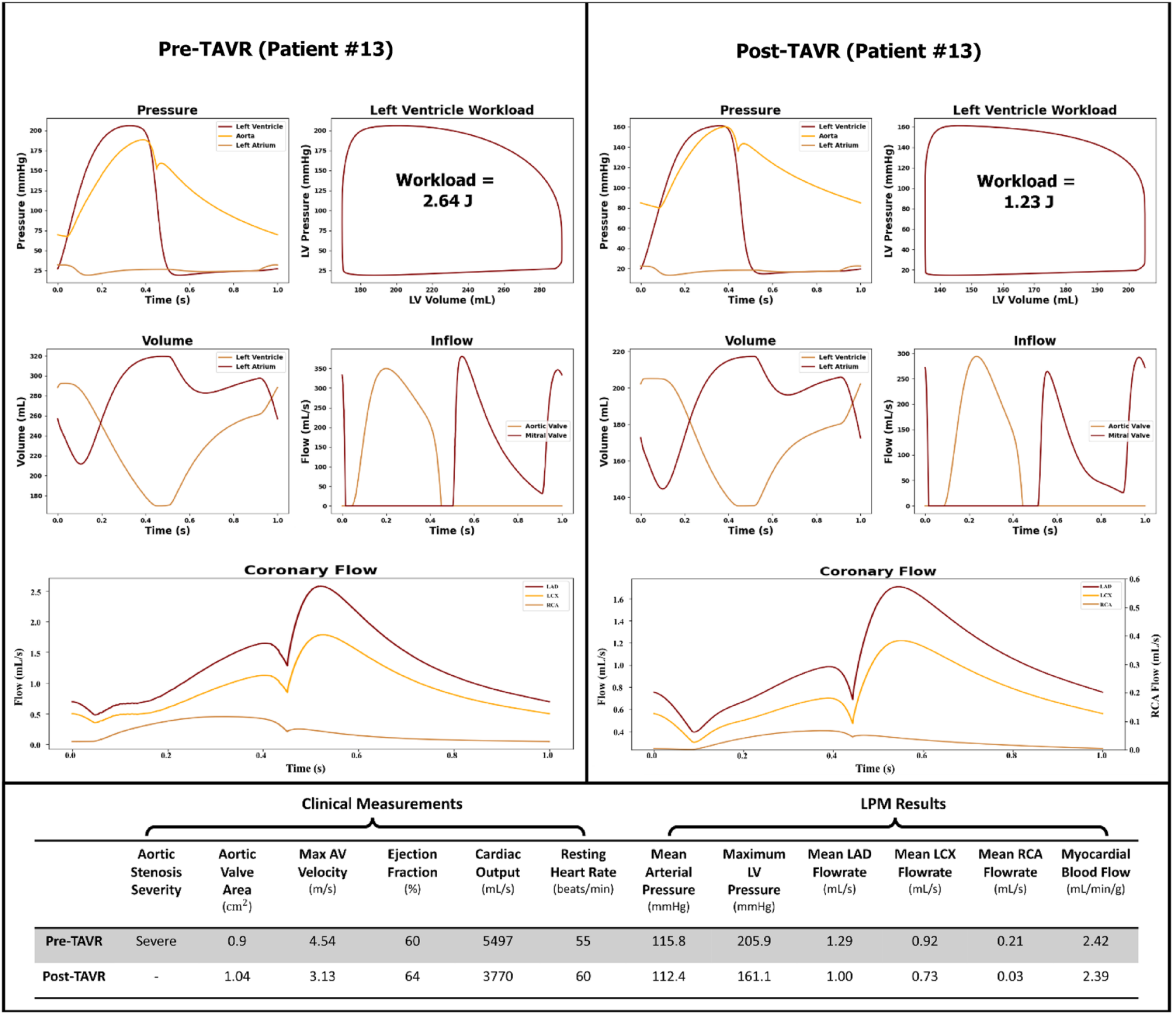
Predicted cardiac and coronary hemodynamics (Patient #13). The plots on the left and right illustrate the pre-TAVR and post-TAVR data respectively.

**Figure 8:**
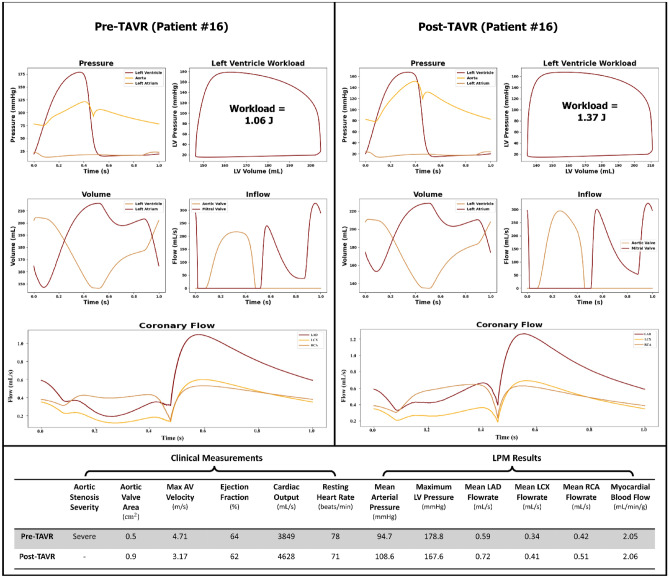
Predicted cardiac and coronary hemodynamics (Patient #16). The plots on the left and right illustrate the pre-TAVR and post-TAVR data respectively.

Figures 6, 7 and 8 illustrate patient specific cardiovascular data for various regions of the heart and coronary arteries in both the pre- and post-TAVR cases. Patients #18, #13 and #16 were selected to illustrate cases in which the procedure led to varying outcomes in coronary flow rates and cardiac dynamics.

Patient #18 was suffering from severe aortic stenosis prior to receiving a TAVR procedure, which had led to an increased burden on the left ventricle. After TAVR, the mean pressure gradient and max aortic valve velocity decreased and all the other predicted hemodynamic metrics increased, including myocardial blood flow. Following TAVR, the aortic valve area increased from 0.80 to 2.30 cm^2^ and the ejection fraction improved from 69 to 71%. Based on the model, the intervention led to an increase in peak LV pressure (+ 4.7%), an increase in the MAP (+ 35.2%), an increase in LV workload (+ 24.0%), an increase in cardiac output (+ 28.3%), and an increase in resting heart rate (+ 2.9%). Overall, an increase in the LAD (+ 31.1%), LCX (+ 32.4%) and RCA (+ 30.8%) flow rates were observed post-TAVR. Additionally, the total estimated myocardial blood flow (coronary blood flow per gram of cardiac mass) increased from 2.36 to 4.06 mL/min/g.

Patient #13 was also suffering from severe aortic stenosis prior to receiving TAVR. After TAVR, the mean pressure gradient, max aortic valve velocity, cardiac output, MAP, max LV pressure and myocardial blood flow all decreased while ejection fraction and resting heart rate increased. Similar to patient #18, the intervention increased the aortic valve area from 0.90 to 1.04 cm^2^ and the ejection fraction increased slightly from 60 to 64%. According to the model, the intervention led to a decrease in the peak LV pressure (− 21.8%), a decrease in LV workload (− 53.4%), a decrease in MAP (− 2.9%), an increase in resting heart rate (+ 9.1%) and a decrease in cardiac output (− 31.4%). The coronary flow in the LAD, LCX and RCA decreased by 22.5%, 20.7% and 85.7% after surgery, respectively. The total estimated myocardial blood flow also decreased from 2.42 to 2.39 mL/min/g.

Patient #16 suffered from the same condition and severity as the other subjects. After TAVR, the mean pressure gradient, max aortic valve velocity, ejection fraction, resting heart rate and max LV pressure decreased while cardiac output and MAP increased. Coronary blood flow rate increased while the overall myocardial blood flow increased slightly after TAVR. As with the other subjects, the aortic valve area increased after TAVR (0.5 to 0.9 cm^2^) and the ejection fraction remained relatively unchanged (64% to 62%). Interestingly though, even though the total coronary flow increased after TAVR (+ 21.5%), the total myocardial blood flow rate barely changed after the surgery (2.05 to 2.06 mL/min/g). This is likely due to the increase in the left ventricle mass index (77 to 93 g/m^2^) which may be a by-product of the increase in left ventricle work (1.06 to 1.37 J).

The examples illustrated by patients #18, #13 and #16 provide insight into the capabilities of the C3VM-LPM to compute patient specific cardiovascular data, including non-invasive insight into the hemodynamics in the coronary arteries. It also further highlights the patient specific nature of treating aortic stenosis and the resulting hemodynamics.

## Discussion

As medicine becomes more patient centered, there is a strong motivation to create patient specific treatment approaches and design tools capable of capturing individual health data^84^. The union between computational science, medical imaging, and cardiology has opened the doors to numerous new patient specific cardiovascular tools. “Cardiology is flow”^85^ and providing clinicians with a non-invasive window into coronary blood flow and global cardiovascular parameters can be advantageous in helping clinicians and cardiologists make treatment decisions^36,86,87^. Furthermore, the development of computationally efficient methods to non-invasively quantify blood flow may be useful in high volume clinical settings.

In this paper, we develop a novel LPM which utilizes non-invasive inputs to simulate blood flow waveforms in the main proximal coronary branches (LAD, LCX and RCA) as well as other global cardiovascular hemodynamic parameters. The model was then applied to 19 patients undergoing TAVR to examine the impact of the procedure on various cardiovascular metrics. The coronary flow results from the computational model were compared with those from a patient specific 3D FSI model (n = 19) and a model sensitivity analysis was conducted.

### Coronary blood flow increase or decrease varies in patients after TAVR

The coronary waveforms from the lumped model for patients with and without aortic stenosis were very consistent with the waveforms reported in literature^70,83,88,89^. For patients without aortic stenosis, a clear bi-phasic flow pattern was present with lower flow during systole and more flow occurring in diastole. In the presence of aortic stenosis, the blood flow during systole decreased considerably (in some cases resulting in zero or negative retrograde flow) and most of the blood flow was delivered to the coronaries in diastole. Garcia et al.^70^, Hongo et al.^88^ and others^83,89^ have observed very similar flow patterns in healthy and aortic stenosis cases.

After TAVR, the model yielded an increase in both the mean and peak flow rates during systole but a decrease during diastole. This trend has been observed in numerous other studies regarding the relationship between coronary flow and TAVR^83,90–92^. In patients with aortic stenosis, systolic blood flow from the ventricle can be limited due to the obstruction caused by the stenotic valve, thus limiting coronary flow. Additionally, the elevated LV pressure enhances the impact of extravascular compression, further restricting systolic coronary flow. After TAVR, the increase in valve orifice area and reduction in ventricular pressure leads to increased blood flow during systole, resulting in an increase in systolic coronary flow. Since aortic stenosis is a systolic phenomenon that impacts the opening rather than closing of the valve, TAVR has been shown to have a smaller impact on the diastolic phases and thus less impact on coronary flow during diastole^90,91^.

This study demonstrated individual differences in terms of coronary blood flow increase or decrease for the full cardiac cycle after TAVR. This varying outcome has been previously noted, for instance, Ben-Dor et al.^83^ found that of the 90 patients in their clinical study, only 48% had a ≥ 10% increase in their left main coronary flow velocity after TAVR. Relative reduction in coronary blood flow has been associated with various negative cardiovascular events including decreased ventricle contractile function, ventricular dysfunction and increased risk of ischemic events^93,94^. Furthermore, moderate or prolonged reduction in coronary blood flow may lead to molecular and morphological changes in the myocardium and may worsen heart failure^93^.

There is currently uncertainty around the optimal management and treatment of AS and coexisting CAD^95^. There is a current debate about whether CAD should be treated before AS, alongside the AS or after the AS^19,91,96^. Additionally, some preliminary research indicates that negative coronary events after TAVR may be driven by impaired coronary flow dynamics and coronary hypoperfusion related to the TAVR prosthesis^19^. Currently clinicians have relatively limited options to examine coronary flow hemodynamics in a rapid and non-invasive fashion^16^. By using computational models like the one presented in this paper, clinicians may eventually be able understand, quantify, and predict adverse coronary related events surrounding TAVR that otherwise might be missed without this additional data and insight.

### Global hemodynamic metrics vary in patients after TAVR

The increase or decrease in certain computed global hemodynamic parameters (LV workload, SBP, DBP and CO) varies from subject to subject after TAVR. LV workload for instance decreased in 10 patients while increasing for 9 subjects after TAVR. While the workload often decreases post-TAVR due to a reduction in afterload^31^, this only occurred in a subset of our subjects. This observed increase in workload may be driven by the interplay of various cardiovascular factors such as the presence of mixed valvular disease including mitral valve regurgitation or post-TAVR complications such as paravalvular leakage (2 patients had mild and moderate to severe PVL respectively)^30^. In regard to blood pressure, according to our model SBP, DBP and MAP all increased in 68% of the subjects. Similarly, Perlman et al.^81^ found that of 150 subjects who underwent TAVR, 51% had sustained increases in blood pressure after the procedure. Interestingly, in this study, subjects with increased blood pressure after TAVR had better a long-term prognosis and fewer adverse events after 30 days and 12 months^81^. While there are some disagreements regarding the benefits and drawbacks of increased blood pressure after TAVR, various studies have found that blood pressure increase or decrease after TAVR is highly dependant on the individual^80^.

Unlike the other global metrics, aortic valve pressure gradient decreased in all but one subject after TAVR. Since implanting the new valve (and increasing the valve effective orifice area) is one of the main aims this procedure, it is expected that the valve pressure gradient would decrease after TAVR^31,41^. In all, due to the highly interconnected and dynamic nature of cardiovascular system, this patient specific variability in global hemodynamics can likely help explain why coronary blood flow increase or decrease after TAVR also varies.

### Limitation of current LPMs to capture coronary blood flow

While there exist some LPMs developed for the coronary arteries^61,62,68,97–100^ very few include patient specific coronary and cardiac segments. In work by Duanmu et al.^68^, the authors use CT images to extract details of the coronary branches to tune the circuit elements but use generic heart functions as inputs to the coronary arteries. Li et al.^61^ also used a similar approach but tuned the model parameters to match generic coronary artery flow patterns for a single patient. Calderan et al.^100^ applied both an in-vivo model and lumped model to characterize the impact of TAVR on coronary artery flow but relied on semi-generic parameters and did not divide the blood flow into the 3 main coronary arteries. Currently, none of the existing pure LPMs can simulate and examine the impact of TAVR on coronary blood flow in a patient specific manner.

In recent years, most of the developments related patient specific computational cardiology models have made use of 3D based modelling (computational fluid dynamics, FSI, Lattice-Boltzmann and others). These powerful models can provide detailed insight into parameters such as wall shear stress, multi-dimensional blood flow patterns, vortical structures and other key parameters. On the other end of the spectrum, LPM offers a simpler and computationally cheaper method to simulate a series of global cardiovascular metrics and flow/pressure waveforms. While LPM sacrifices the ability to compute many of the 3D based parameters, it reduces the computational time from hours or days to seconds and makes model automation easier.

## Limitations

This study was performed and validated using 19 patients and showed strong agreement with both the pre- and post-TAVR results from the 3D FSI models. Future studies must be conducted on a larger population of patients (both pre- and post-TAVR) with a considerable inter- and intra-patient variability with a broad range of diseases to further confirm the clinical findings of this study. Numerous FSI models have been previously shown to accurately simulate blood flow in the cardiovascular system, including the coronary arteries^26,60,69,101^. Furthermore, the cardiac LPM was previously designed to capture complex valvular, vascular, and ventricular diseases and has been previously validated against 49 patients with a wide range of diseases^41^. Nevertheless, future studies must consider validating the coronary flow waveforms from the model for both systole and diastole phases against invasive coronary catheter data.

As outlined in the analysis, the model is relatively sensitive to changes in coronary vessel cross sectional area, which is currently computed from 3D CT-based reconstructions. While a standard segmentation and reconstruction process is applied to all patients, it is currently done manually and is prone to a small degree of human error. This error could be reduced by using coronary CT angiography or standard angiography which produce higher quality coronary images.

The model is also sensitive to changes in mean arterial pressure, an input parameter currently obtained using a clinical grade sphygmomanometer. As coronary perfusion pressure (which is partially based on MAP) increases in healthy patients, the body naturally adjusts coronary resistance to help regulate the coronary blood flow rate^77^. Although it is not fully clear how this autoregulation is impacted by the presences of AS and CAD and only occurs for range of pressures, future models could be enhanced through the addition of patient specific control loops to further regulate the relationship between coronary pressure and flow.

## Supplementary Information

Supplementary Information.
