# Supplementary material for: Impact of TAVR on coronary artery hemodynamics using clinical measurements and image‐based patient‐specific in silico modeling
Source: Sci Rep. 2023 Jun 2;13:8948. doi: 10.1038/s41598-023-31987-w (PMC10238523; doi:10.1038/s41598-023-31987-w)
Supplement: Supplementary file 1 — Supplementary Information. [file 41598_2023_31987_MOESM1_ESM.docx]

**Supplemental Material**


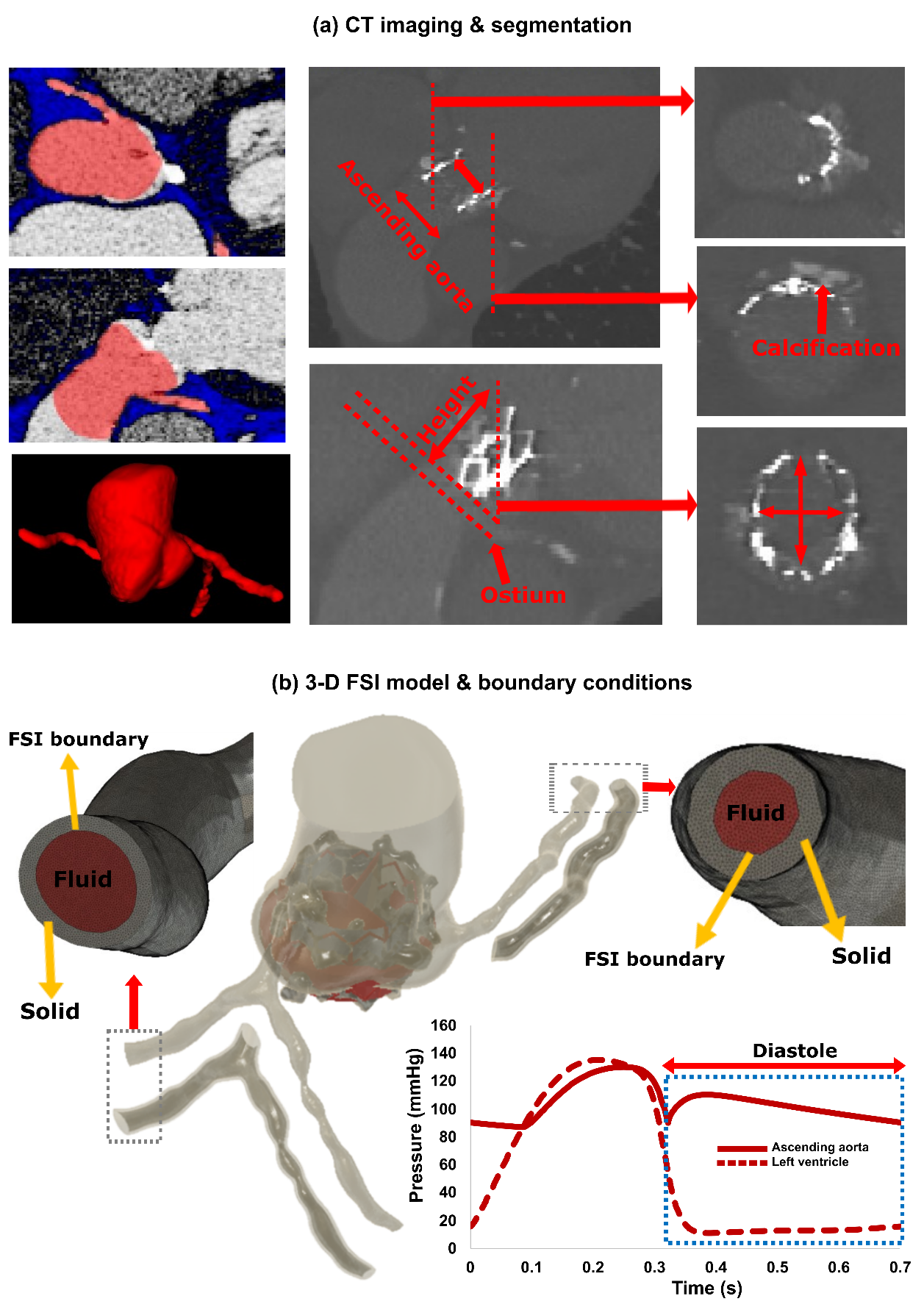


**Figure S1. Computational domain and boundary conditions of 3D FSI model**


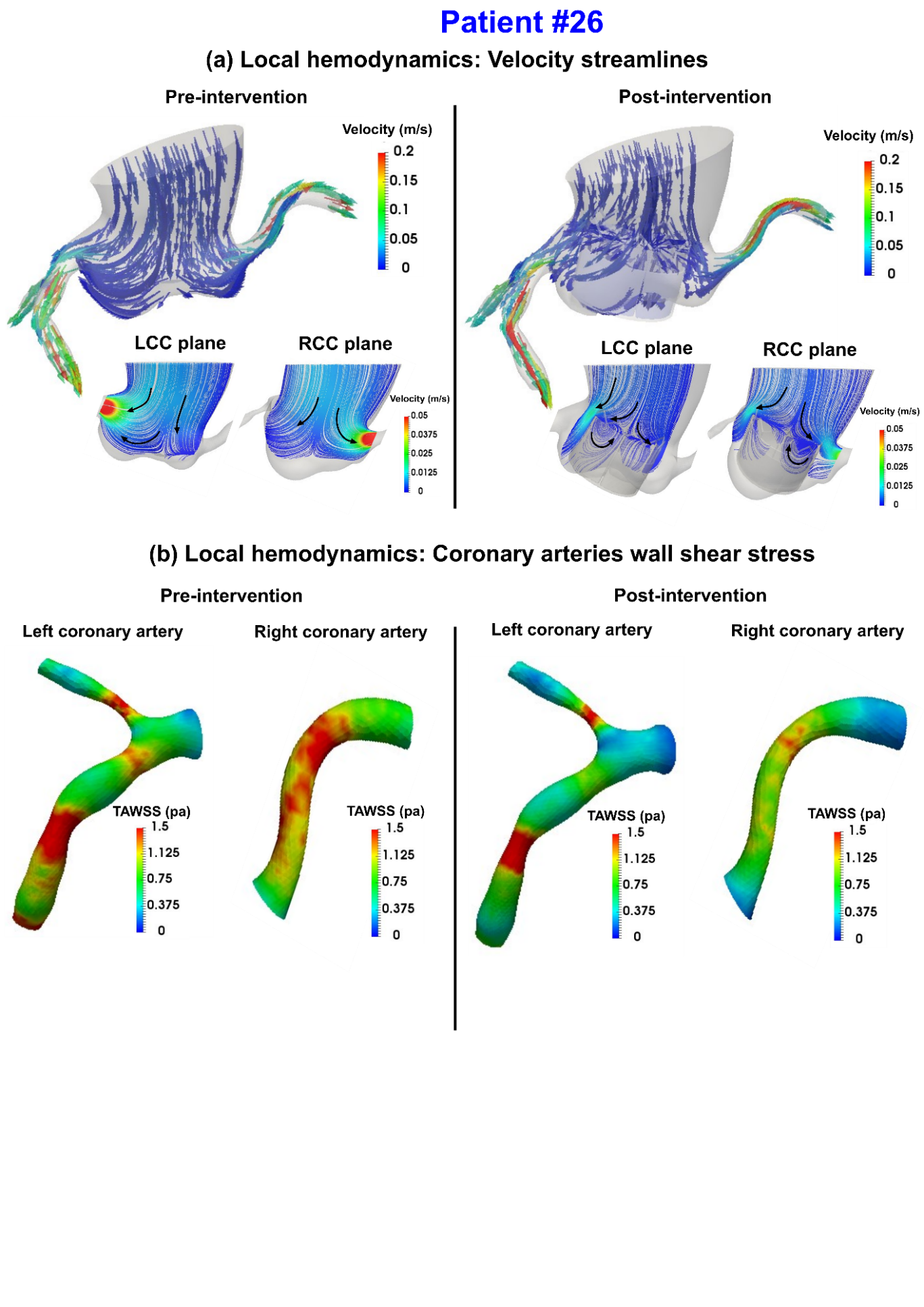


**Figure S2. 3D flow result sample of coronary arteries and aortic root obtained from 3D FSI model**

**Table S1. Coronary vessel segmentation sensitivity analysis**

| **Mean Predicted Coronary Flow Rate (mL/s) Across the Full Cardiac Cycle** | | | |
| --- | --- | --- | --- |
| **LCX Diameter (mm)** | **LCX Flow Rate** | **LAD Flow Rate** | **RCA Flow Rate** |
| 1.0 | 0.032 | 1.245 | 1.030 |
| 1.5 | 0.087 | 1.203 | 0.989 |
| 2.0 | 0.181 | 1.175 | 0.979 |
